# ^18^F-MK-6240 uptake in cortical tau and hemorrhagic lesions in a case of Alzheimer’s disease with possible crossed aphasia

**DOI:** 10.1016/j.ensci.2025.100596

**Published:** 2025-11-21

**Authors:** Masaki Ikeda, Kenji Ishibashi, Masakuni Amari, Masaru Matsumura, Hiroo Kasahara, Jun Toyohara, Sayaka Kodaira, Tetsuya Higuchi, Yoshio Ikeda, Yoshito Tsushima, Koichi Okamoto, Kenji Ishii, Masamitsu Takatama

**Affiliations:** a Division of General Education (Neurology), Faculty of Health & Medical Care, Saitama Medical University, 1397-1 Yamane, Hidaka, Saitama 350-1241, Japan; b Department of Neurology, Geriatrics Research Institute and Hospital, 3-26-8 Otomo machi, Maebashi, Gunma 371-0847, Japan; c Diagnostic Neuroimaging Research, Research Team for Neuroimaging, Tokyo Metropolitan Institute for Geriatrics and Gerontology, 35-2 Sakae-cho, Itabashi-ku, Tokyo 173-0015, Japan; d Department of Neuroscience and Neurosurgery, Central Gunma Neurosurgery Hospital, 64-1, Nakao-machi, Takasaki, Gunma 370-0001, Japan; e Department of Neurology, Gunma University Graduate School of Medicine, 3-29-22 Showa-machi, Maebashi, Gunma 371-8511, Japan; f Department of Diagnostic Radiology and Nuclear Medicine, Gunma University Graduate School of Medicine, 3-29-22 Showa-machi, Maebashi, Gunma 371-8511, Japan; g Department of Rehabilitation, Shibukawa Chuo Hospital, 508-1, Ishihara, Shibukawa, Gunma 370-0007, Japan

**Keywords:** Alzheimer’s disease, Cerebral amyloid angiopathy, Tau, ^18^F-MK-6240, ^18^F-THK5351, Positron emission tomography

## Abstract

We report neuroimaging findings from a 74-year-old right-handed male with Alzheimer’s disease (AD) and lesions of cerebral amyloid angiopathy (CAA), utilizing ^11^C-PiB-PET, ^18^F-THK5351-PET, and ^18^F-MK-6240-PET. ^11^C-PiB-PET showed positive findings consistent with AD. ^18^F-THK5351 accumulated in regions of astrogliosis due to tau pathology, subcortical hemorrhage, cortical superficial siderosis (cSS), and monoamine oxidase-B rich areas. ^18^F-MK-6240 accumulated in regions with tau pathology, subcortical hemorrhage, and cSS, but not notably in CAA-related microbleeds (CMBs). ^99m^Tc-ECD SPECT, conducted 9 years post-diagnosis, revealed reduced cerebral blood flow in the bilateral lower temporal lobes and the right posterior temporo-parietal lobes, overlapping the subcortical hemorrhage and cSS. The patient exhibited progression of global cognitive decline and persistent word fluency deficits (name listing) on neuropsychological examination from the early stage of the disease, irrespective of the right hemorrhagic lesions in the non-dominant hemisphere, suggesting possible crossed aphasia. This is the first report of ^18^F-MK-6240 binding to a subcortical hemorrhage and cSS lesions, highlighting its binding differences compared to smaller vascular leakages, such as CMBs due to CAA. These results may help refine PET imaging interpretation and diagnostic accuracy for AD with concurrent CAA.

## Highlights

• ^18^F-MK6240 accumulated in tau, subcortical hemorrhage, and superficial siderosis.
• No uptake was observed in cerebral microbleeds.
• This case exhibited progression of cognitive decline with possibly crossed aphasia.

## 1 Introduction

Alzheimer’s disease (AD) is characterized by pathological hallmarks such as senile plaques and cerebral amyloid angiopathy (CAA), both associated with amyloid beta (Aβ) deposition, alongside neurofibrillary tangles resulting from tau accumulation [1]. ^18^F-MK-6240 is a validated neuroimaging biomarker for the detection of tau pathology in AD, aiding in distinguishing AD from non-AD four-repeat tauopathies such as progressive supranuclear palsy and corticobasal degeneration [[2], [3]]. Another tracer, ^18^F-THK5351, binds to monoamine oxidase-B (MAO-B) [4], facilitating the identification of astrogliosis in various neurodegenerative diseases [5] and in cases of cerebral infarction [6]. Furthermore, it can detect astrogliosis related to subcortical hemorrhages and cortical superficial siderosis (cSS) in AD with CAA lesions [7]. ^18^F-MK-6240 also shows off-target binding to neuromelanin and melanin-containing cells and demonstrates a weak but measurable amount of affinity for hemorrhagic parenchymal brain tissue in postmortem analyses [8,9]; however, this has not yet been reported *in vivo*.

## 2 Case presentation

A 74-year-old right-handed male experienced recent memory impairment 2 years earlier and underwent assessment at the Geriatric Research Institute and Hospital (Maebashi, Gunma, Japan) at X years/4 months. The scores were as follows: Mini-Mental State Examination (MMSE) 27/30, Hasegawa’s Dementia Scale-Revised (HDS-R) 22/30, Montreal Cognitive Assessment (MoCA) 18/30, and Frontal Assessment Battery (FAB) 10/18. The neuropsychological evaluation demonstrated deficits in time orientation, recent memory, calculation, and object recall (MMSE, HDS-R, MoCA), with a particular difficulty in word fluency (listing vegetable names) in the HDS-R, while verbal comprehension and fluent speech were preserved. Over the following years, cognition further deteriorated, with MMSE and HDS-R declining to 10/30 by X + 9 years/4 months. The patient had no psychiatric symptoms or prior incidents of stroke or head trauma.

**Fig. 1:**
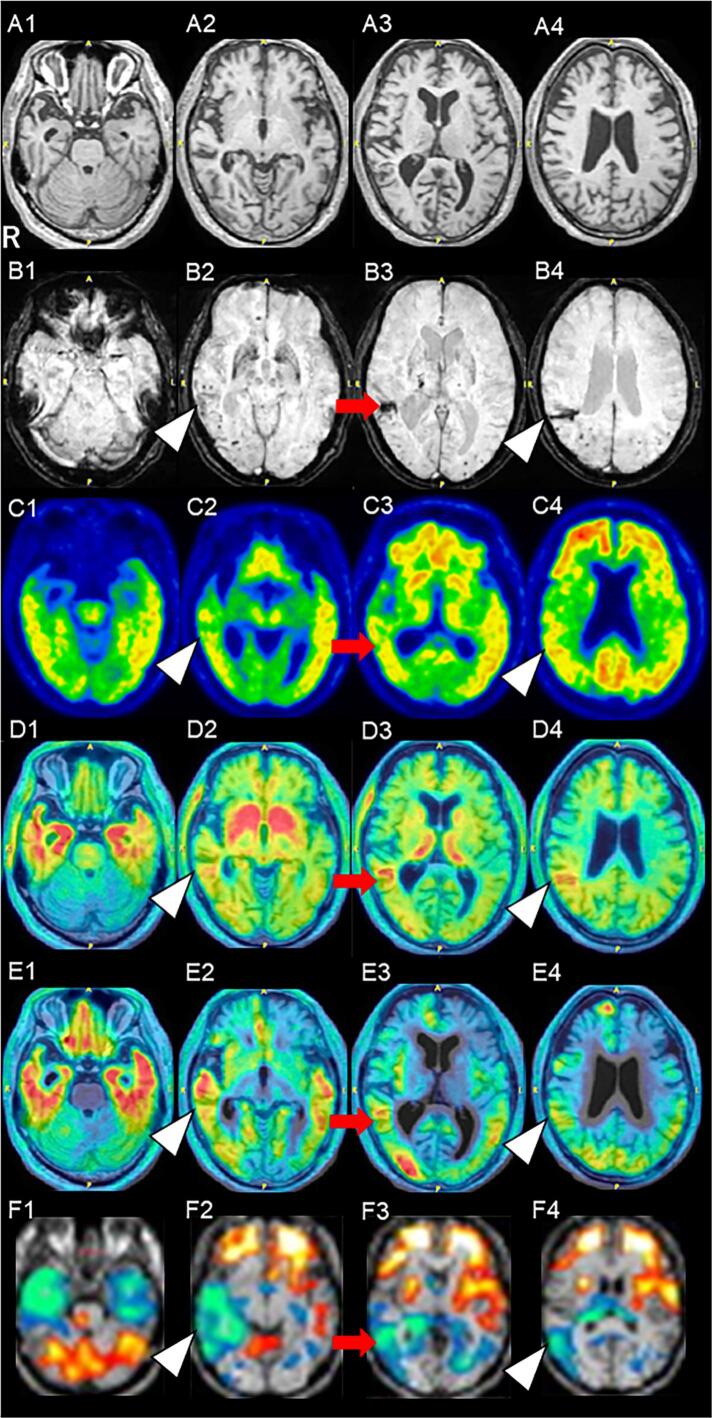
Neuroimaging. T1-weighted MRI (T1WI) (X/04). B. SWI MRI (X + 2/04). ^11^C-PiB-PET (X/07). D. ^18^F-THK5351-PET (X + 2/11) superimposed on T1WI MRI. E. ^18^F-MK-6240-PET (X + 9/10) superimposed on MRI T1WI. F. ^99m^Tc ECD-SPECT (eZIS) (X + 9/02). Sections shown A1–F4: transverse view.

T1-weighted magnetic resonance imaging (T1WI MRI) (X/04) revealed dilation of the inferior horn of the lateral ventricle and Sylvian fissure, accompanied by bilateral anterior and medial temporal lobe atrophy, more marked on the right (Fig. 1A1), as well as diffuse atrophy in the frontal, temporal, and parietal cortices (Fig. 1A2–A4). Susceptibility-weighted imaging (SWI MRI) (X + 2/04) demonstrated subcortical hemorrhage (arrow, Fig. 1B3) and cSS in the right temporal and parietal cortices (arrowheads, Fig. 1B2, B4). Multiple cerebral microbleeds (CMBs) were detected bilaterally in the temporal and occipital cortices (Fig. 1B2–B4). ^11^C-Pittsburgh compound B positron emission tomography (^11^C-PiB-PET) (X/07) showed hyperintensities in the bilateral posterior cingulate, precuneus, and frontal, temporal, and parietal cortices, consistent with AD (Fig. 1C1–C4).

PET with ^18^F-THK5351 (X + 2/11) superimposed on T1WI MRI demonstrated significant tracer uptake in the bilateral medial and lateral temporal cortices and the bilateral frontal cortices, reflecting astrogliosis secondary to tau accumulation (Fig. 1D1, D2). This tracer accumulation was also observed in the right lateral temporal lobe and parietal cortex at the sites of subcortical hemorrhage (arrow, Fig. 1D3) and cSS (arrowheads, Fig. 1D2, D4), indicative of astrogliosis associated with CAA pathology. Additional accumulation was noted in the bilateral globus pallidus, caudate nucleus, and thalamus, consistent with MAO-B binding (Fig. 1D2, D3).

^18^F-MK-6240-PET imaging (X + 9/09) superimposed on T1WI MRI, revealed hyperintensities in bilateral medial temporal cortices, straight gyri of the frontal lobe, lateral temporal, occipital, and parietal cortices, indicating tau accumulation (Fig. 1E1–E4). Notably, ^18^F-MK-6240 uptake was seen in the right temporal lobe encompassing the subcortical hemorrhage (arrow, Fig. 1E3) and cSS (arrowheads, Fig. 1E2, E4), but not in the CMBs (Fig. 1E2–E4). ^99m^Tc-ECD-SPECT (eZIS) (X + 9/02) showed hypoperfusion in the bilateral medial temporal lobes and temporal poles (Fig. 1F1), as well as in the posterior regions of the right temporal lobe and the parietal cortices that included corresponding regions of the subcortical hemorrhage (arrow, Fig. 1F3) and cSS (arrowheads, Fig. 1F2, F4).

**Fig. 2:**
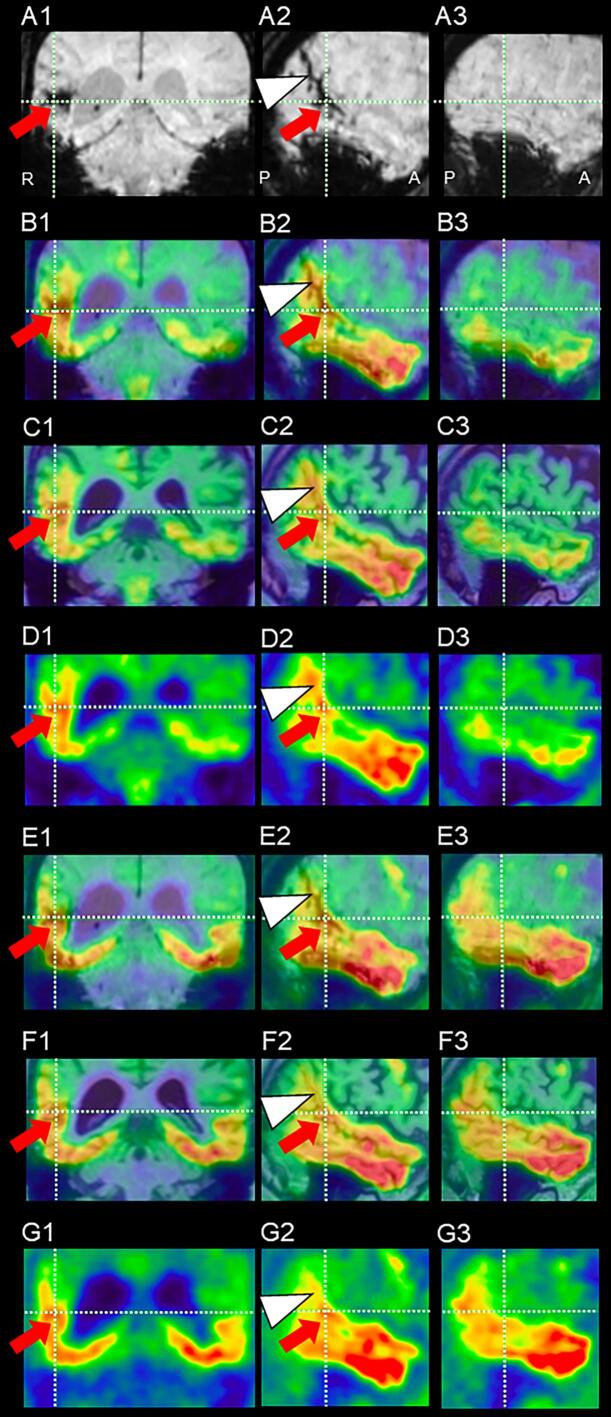
^18^F-THK5351 and ^18^F-MK-6240 positron emission tomography (PET). A. MRI SWI (X + 2/04). B–D. F-THK5351-PET (X + 2/11). E–G ^18^F-MK-6240-PET (X + 9/10). A1–G1: coronal view. A2–G2, A3–G3: sagittal view.

SWI MRI demonstrated a distinct area of low-intensity consistent with subcortical hemorrhage in the right posterior superior temporal gyrus, located at the intersection of the vertical and horizontal reference lines (arrows, Fig. 2A1, A2), and cSS in the right angular gyrus and parietal cortex (arrowhead, Fig. 2A2). No hemorrhage or cSS was observed on the left (Fig. 2A1, A3). Several CMBs appeared in both occipital cortices (Fig. 2A2, A3).

^18^F-THK5351-PET showed significant uptake in the bilateral lower temporal cortices with right-side dominance (Fig. 2B1, B2, B3) and the right temporal subcortical region, located at the intersection of the vertical and horizontal reference lines (arrows, Fig. 2B1, C1, D1; Fig. 2B2, C2, D2), indicative of astrogliosis due to the subcortical hemorrhage (arrow, Fig. 2A1, A2) and the cSS (arrowhead, Fig. 2A2) in the SWI MRI. No comparable uptake was observed in the corresponding left middle-superior temporal lobe (Fig. 2B1, C1, D1; B3, C3, D3). ^18^F-THK5351-PET revealed extensive accumulation in the right hemisphere. In contrast, ^18^F-THK5351 uptake in the left hemisphere was limited to the inferior temporal gyrus and temporal pole and was considerably weaker, with no uptake corresponding to the right subcortical hemorrhage (arrows, Fig. 2B3, C3, D3) and cSS (arrowheads, Fig. 2B3, C3, D3).

^18^F-MK-6240-PET showed tracer accumulation in the bilateral inferior and middle temporal gyri, indicative of tau accumulation, and in the right superior temporal gyrus corresponding to the subcortical hemorrhage identified at the intersection of the vertical and horizontal reference lines (arrows, Fig. 2E1, F1, G1). However, no accumulation was observed in the corresponding area on the left. This imaging also showed widespread tracer accumulation across the bilateral superior, middle, and inferior temporal gyri (Fig. 2E2, F2, G2; E3, F3, G3), reflecting tau accumulation, as well as in the right posterior superior temporal gyrus and angular gyrus, aligning with the subcortical hemorrhage at the intersection of the vertical and horizontal reference lines (arrows, Fig. 2E2, F2, G2) and the cSS lesions (arrowheads, Fig. 2E2, F2, G2). No ^18^F-MK-6240 accumulation was observed in the left hemisphere corresponding to the right (Fig. 2E3, F3, G3). At X + 3 years and 8 months, he presented a low MMSE score of 22 and demonstrated difficulty in “word fluency (listing vegetable names)”, scoring 18 on the HDS-R. At the same time, the standard language test of aphasia (SLTA) showed low scores, particularly in word retrieval tasks: “Word fluency (listing)” and “Explain picture story” (Table 1). Persistently low scores in “Word fluency” on the HDS-R have been confirmed since the first examination to date.

**Table 1:** Result of the standard language test of aphasia (SLTA).

Functions	No.	Items	Correct answer (%)
I. Auditory comprehension	1	Auditory comprehension of words	70
	2	Auditory comprehension of short sentences	70
	3	Follow verbal commands	60
	4	Auditory comprehension of Kana letter	90
II. Speaking	5	Speak object naming	70
	6	Word repetition	100
	7	Explain behavior in pictures	70
	8	Verbal explanation of picture story	40
	9	Sentence repetition	90
	10	Verbal fluency (listing)	33.3
	11	Read aloud Kanji words	80
	12	Read aloud Kana letters	100
	13	Read aloud Kana words	80
	14	Read aloud short sentences	60
III. Reading comprehension	15	Read Kanji word-picture matching	70
	16	Read Kana word-picture matching	90
	17	Read short sentence-picture matching	70
	18	Follow written commands	60
IV. Writing	19	Write Kanji words	50
	20	Write Kana words	60
	21	Narrative writing	50
	22	Dictate Kana letters	70
	23	Dictate Kanji words	50
	24	Dictate Kana words	60
	25	Dictate short sentences	40
V. Calculation	26	Calculation	50

SLTA was assessed at X + 3 years and 8 months, with underlines showing less than 50% correct answers (%).

## 3 Discussion

This case report describes a patient with AD and CAA-related lesions, including subcortical hemorrhage and cSS in the right temporal, angular gyrus, and parietal lobes, as well as multiple microbleeds in both occipital cortices.

^18^F-THK5351 exhibited uptake in both medial and lateral temporal cortices, reflecting astrogliosis associated with tau deposition. Additional uptake was seen in the right subcortical hemorrhage and cSS regions affected by CAA, with no similar findings on the opposite side. By contrast, ^18^F-MK-6240 demonstrated extensive accumulation across the bilateral superior, middle, and inferior temporal gyri, with increased uptake in the right posterior superior temporal gyrus and angular gyrus at the location of the subcortical hemorrhage and cSS. These observations suggest that ^18^F-MK-6240 may be localized to regions of tau pathology and possibly to parenchymal hemorrhage and cSS, although it does not bind to CMBs. While ^18^F-MK-6240 is a validated marker for tau pathology in AD, it is also characterized by off-target binding to neuromelanin and melanin-containing cells and weak but detectable binding to hemorrhagic tissue in postmortem analyses, but not to vascular Aβ in blood vessel walls [8,9]. Our case emphasizes the utility of ^18^F-MK-6240-PET in detecting not only tau pathology but also the subcortical hemorrhage and cSS, while ^18^F-THK5351-PET identifies astrogliosis related to tau pathology, subcortical hemorrhage, and cSS, as reported previously [7]. Pathologically, astrogliosis appears markedly alongside cSS lesions [10]. In this patient, ^18^F-MK-6240 detected the subcortical hemorrhage and cSS, likely attributable to off-target binding with blood products in parenchymal tissues; however, it seems not to bind to vascular Aβ and/or CMBs, which represent only a small amount of blood leakage into perivascular spaces. In this case, the CAA lesions of the right posterior superior temporal gyrus and angular gyrus might be associated with the word retrieval difficulties in “Word fluency” and “Explain picture story” on the SLTA, as well as persistent impairment in “Word fluency” on the HDS-R, suggesting possible crossed aphasia.

This finding of the case has some limitations in the tracer’s clinical applicability and may confound image interpretation in AD patients with concurrent cerebrovascular disease. In diagnosis of neuroimaging in MK-6240-PET examination, we may need to pay additional attention and take a cautious attitude to high signal lesions of MK6240 accumulation in central nervous system, whether reflecting tau accumulation or hemorrhagic lesions as off-target binding in clinical settings. We experienced only one AD patient by MRI (SWI) and MK-6240-PET examinations, to confirm this finding, it would be required to analyze a large study of AD patients with hemorrhagic CAA lesions, i.e., subcortical hemorrhage, cSS, and lobar CMBs, using MRI (SWI or T2*WI), amyloid PET, and MK-6240-PET examinations. At this moment, we should recognize this limitation with the neuroimaging finding because of one-single case and require clarifying the practical relevance (or cautionary implication) of this finding in MK-6420-PET examination.

## 4 Conclusions

This is the first report documenting a patient with AD, subcortical hemorrhage, and cSS due to CAA, showing ^18^F-MK-6240 accumulation in cortical tau pathology, in addition to subcortical hemorrhage and cSS as off-target effects, without corresponding uptake in CMBs. These findings may enhance the diagnostic value of ^18^F-MK-6240-PET imaging and contribute to improved accuracy in the clinical diagnosis of AD.

## CRediT authorship contribution statement

**Masaki Ikeda:** Writing – original draft, Supervision, Methodology, Conceptualization. **Kenji Ishibashi:** Methodology, Investigation. **Masakuni Amari:** Writing – review & editing, Data curation. **Masaru Matsumura:** Investigation. **Hiroo Kasahara:** Investigation. **Jun Toyohara:** Investigation. **Sayaka Kodaira:** Data curation. **Tetsuya Higuchi:** Data curation. **Yoshio Ikeda:** Data curation. **Yoshito Tsushima:** Data curation. **Koichi Okamoto:** Writing – review & editing. **Kenji Ishii:** Data curation. **Masamitsu Takatama:** Writing – review & editing.

## Ethical statement

This case report received approval from the Ethics Committee of the Tokyo Metropolitan Institute for Geriatrics and Gerontology and the Geriatrics Research Institute and Hospital. Informed consent was obtained from the patient and his family prior to publication.

## Funding

This research did not receive any specific grant from funding agencies in the public, commercial, or not-for-profit sectors.

## Declaration of competing interest

None.

## References

[1] Jellinger J.A. (2022). Recent update on the heterogeneity of the Alzheimer’s disease spectrum. J. Neural Transm. (Vienna).

[2] Ishibashi K., Kurihara M., Toyohara J., Ishii K., Iwata A. (2024). Pitfalls of amyloid-beta PET: comparisons with ^18^F-MK-6240 and ^18^F-THK5351 PET. Clin. Nucl. Med..

[3] Malarte M.L., Gillberg P.G., Kumar A., Bogdanovic N., Lemoine L., Nordberg A. (2023). Discriminative binding of tau PET tracers PI2620, MK6240 and RO948 in Alzheimer’s disease, corticobasal degeneration and progressive supranuclear palsy brains. Mol. Psychiatry.

[4] Ng K.P., Pascoal T.A., Mathotaarachchi S., Therriault J., Kang M.S., Shin M., Guiot M.C., Guo Q., Harada R., Comley R.A., Massarweh G., Soucy J.P., Okamura N., Gauthier S., Rosa-Neto P. (2017). Monoamine oxidase B inhibitor, selegiline, reduces 18F THK5351 uptake in the human brain. Alzheimers Res. Ther..

[5] Ishibashi K. (2024). Clinical application of MAO-B PET using (18)F-THK5351 in Neurological disorders. Geriatr. Gerontol. Int..

[6] Ishibashi K., Kameyama M., Tago T., Toyohara J., Ishii K. (2017). Potential use of 18F-THK5351 PET to identify Wallerian degeneration of the pyramidal tract caused by cerebral infarction. Clin. Nucl. Med..

[7] Ikeda M., Okamoto K., Suzuki K., Takai E., Kasahara H., Furuta N., Furuta M., Tashiro Y., Shimizu C., Takatama S., Naito I., Sato M., Sakai Y., Takahashi M., Amari M., Takatama M., Higuchi T., Tsushima Y., Yokoo H., Kurabayashi M., Ishibashi S., Ishii K., Ikeda Y. (2021). Recurrent lobar hemorrhages and multiple cortical superficial siderosis in a patient of Alzheimer’s disease with homozygous *APOE* ε2 allele presenting hypobetalipoproteinemia and pathological findings of ^18^F THK5351 positron emission tomography: a case report. Front. Neurol..

[8] Aguero C., Dhaynaut M., Normandin M.D., Amaral A.C., Guehl N.J., Neelamegam R., Marquie M., Johnson K.A., El Fakhri G., Frosch M.P., Gomez-Isla T. (2019). Autoradiography validation of novel tau PET tracer [F-18]-MK-6240 on human postmortem brain tissue. Acta Neuropathol. Commun..

[9] Aguero C., Dhaynaut M., Amaral A.C., Amaral A.C., Moon S.H., Neelamegam R., Scapellato M., Carazo-Casas C., Kumar S., El Fakhri G., Johnson K., Frosch M.P., Normandin M.D., Gómez-Isla T. (2024). Head-to-head comparison of [18F]-Flortaucipir, [18F]-MK-6240 and [18F]-PI-2620 postmortem binding across the spectrum of neurodegenerative diseases. Acta. Neuropathol..

[10] Auger C.A., Perosa V., Greenberg S.M., van Veluw S.J., Kozberg M.G. (2023). Cortical superficial siderosis is associated with reactive astrogliosis in cerebral amyloid angiopathy. J. Neuroinflammation..

